# The Fecal Microbial Community of Breast-fed Infants from Armenia and Georgia

**DOI:** 10.1038/srep40932

**Published:** 2017-02-02

**Authors:** Zachery T Lewis, Ketevan Sidamonidze, Vardan Tsaturyan, David Tsereteli, Nika Khachidze, Astghik Pepoyan, Ekaterine Zhgenti, Liana Tevzadze, Anahit Manvelyan, Marine Balayan, Paata Imnadze, Tamas Torok, Danielle G. Lemay, David A. Mills

**Affiliations:** 1 Department of Food Science and Technology, University of California, Davis, CA, USA; 2 Foods for Health Institute, University of California, Davis, CA, USA; 3 Department of Viticulture and Enology, University of California, Davis, CA, USA; 4 National Center for Disease Control and Public Health of Georgia, Tbilisi, Georgia; 5 IAHAHI (International Association for Human and Animals Health Improvement), Yerevan, Armenia; 6 Armenian National Agrarian University, Yerevan, Armenia; 7 Earth Sciences Division, Lawrence Berkeley National Laboratory, Berkeley, CA, USA; 8 Genome Center, University of California, Davis, CA, USA

## Abstract

Multiple factors help shape the infant intestinal microbiota early in life. Environmental conditions such as the presence of bioactive molecules from breast milk dictate gut microbial growth and survival. Infants also receive distinct, personalized, bacterial exposures leading to differential colonization. Microbial exposures and gut environmental conditions differ between infants in different locations, as does the typical microbial community structure in an infant’s gut. Here we evaluate potential influences on the infant gut microbiota through a longitudinal study on cohorts of breast-fed infants from the neighboring countries of Armenia and Georgia, an area of the world for which the infant microbiome has not been previously investigated. Marker gene sequencing of 16S ribosomal genes revealed that the gut microbial communities of infants from these countries were dominated by bifidobacteria, were different from each other, and were marginally influenced by their mother’s secretor status. Species-level differences in the bifidobacterial communities of each country and birth method were also observed. These community differences suggest that environmental variation between individuals in different locations may influence the gut microbiota of infants.

The time period directly following birth is a critical developmental window where there is a need to both protect the vulnerable infant from disease, and to educate the neonatal immune system^1^,^2^,^3^. Dysbiosis, an imbalanced pattern of microbiota composition or colonization during early development, is associated with a number of disease states including obesity, metabolic syndrome, chronic inflammatory bowel diseases, nonalcoholic steatohepatitis, atherosclerosis, type 1 diabetes, allergy, asthma, celiac disease, kwashiorkor, autism, atopy, and other autoimmune diseases (reviewed in refs 4 and 5). The structure of an infant’s intestinal microbiome is influenced both by the selective environmental conditions in the gut such as carbohydrate availability, pH, activity of the immune system and the arrival of bacteria into the ecosystem due to the sources of microbes to which an infant is exposed. A mechanism by which a mother could beneficially influence the microbiota of her infant via manipulation of the environmental conditions in the gut or the control of microbial exposures may help prevent dysbiosis, and thus be evolutionarily advantageous^5^.

Breast milk has been shaped over mammalian evolution to promote the growth and development of infants. Non-digestible sugars in breast milk, known as human milk oligosaccharides (HMOs), are beneficial to the infant in a number of ways, such as providing protection from pathogens^6^,^7^,^8^. HMOs can be bound to other compounds in milk as glycoconjugates, which are known as human milk glycans (HMGs)^7^. HMGs may play a similar role to free HMOs^9^. These maternally-provided glycans are hypothesized to guide the assembly of the microbial community in the gut due to the ability of only select microbial species to metabolize them^7^,^10^. Bifidobacteria are often the major HMG-consuming constituent of the gut microbiota in breast-fed infants^11^,^12^,^13^,^14^,^15^,^16^, and may provide unique benefits to the newborn, including reducing inflammation, improving gut permeability, and improving responses to both oral and parenteral vaccines^17^,^18^,^19^,^20^,^21^,^22^. Specific analysis of infant-borne bifidobacteria clearly demonstrate that certain species and strains possess a unique genetic capacity to consume HMOs and HMGs and *B. longum* subsp. *infantis (B. infantis*) in particular is the only species shown to consume the complete constellation of HMO/Gs present in milk^9^,^23^. We have recently shown how the genetic capacity of the mother, specifically the FUT2 gene status (also known as secretor status), can influence the bifidobacterial colonization phenotype^24^. Thus, colonization of the infant gut is driven by a mother’s genetics (i.e. expressed milk glycome), infant environmental exposure, and the genetic makeup of the bifidobacteria transferred to the infant—components of which appear to have co-evolved over mammalian history^25^.

However, due to the observed differences in the microbiota of breast fed infants (especially in bifidobacterial abundances) from different countries (Norway^26^, Sweden^27^, Canada^28^, Italy^29^, Switzerland^30^, Bangladesh^22^, the USA^31^, Malawi and Finland^32^) we sought to examine the influences on the microbiome of infants from an area of the world that has not previously been studied. The present study investigates country of origin and birth method (differential sources of microbial exposure) and mother’s secretor-status (an influence on environmental conditions) as possible factors influencing the infant gut microbiome in two cohorts, one from Yerevan, Armenia and one from Tbilisi, Georgia. These countries, like any area on earth, have unique exposure patterns and environmental conditions, however their proximity to one another allows us to test the effect of national boundaries while maintaining relatively low separation by geographic distance.

## Methods

### Sample collection

Fecal samples were collected from exclusively breastfed healthy infants at three time points, one week of life, one month of life, and 3 months of life. Exclusion criteria included formula or other supplemental feeding, mothers who received antibiotic treatments in the year before enrollment, and infants receiving antibiotic treatment. Parents were provided with 50 ml tubes and wooden tongue depressors to collect infant feces. Samples were delivered to the laboratory no more than 2 hours after collection. Metadata questionnaires were filled out at the subject’s home before obtaining samples. Informed consent was obtained from all mothers while they were in the maternity ward. The study protocol was approved by an institutional review board at the Georgian Maternal and Child Care Union (IRB approval #2011-009), and by the Ethics Committee at the Ministry of Education and Science of Armenia (ISTC Project #A-1957) and the Armenian Ministry of Health (M2/938-14 06.02.2014) and all methods were carried out in accordance with the approved guidelines.

### DNA Extraction

DNA was extracted using the ZR Fecal DNA Miniprep Kit (Zymo Research Irvine, CA) with a bead-beating step as per manufacturer’s instructions^33^.

### Sequencing and Analysis

#### Illumina sequencing—V4 region

Extracted DNA was prepared for marker gene sequencing as previously described^34^ with the following modifications. The following universal barcoded primers were used to PCR amplify the V4 region of the 16S rRNA gene, and Illumina sequencing adapters were subsequently ligated to the amplicons (adapters are italicized and an example barcode is highlighted in bold): V4F (5′-*AATGATACGGCGACCACCGAGATCTACACTCTTTCCCTACACGACGCTCTTCCGATCT* **ACTGCTGA**GTGTGCCAGCMGCCGCGGTAA-3′) and V4Rev (5′-*CAAGCAGAAGACGGCATACGAGATCGGTCTCGGCATTCCT GCTGAACCGCTCTTCCGATCT*CCGGACTACHVGGGTWTCTAAT-3′)^34^. PCRs were heated to 94 °C for 3 min to denature the DNA, amplified for 25 cycles of 94 °C for 45 s, 50 °C for 60 s, and 72 °C for 90 s, after which a final extension of 10 min at 72 °C was added to ensure complete amplification. PCR reactions contained 7.5 μl 2x GoTaq Green Master Mix (Promega, Madison, WI), 0.6 μl 25 mM MgCl_2_, 3.6 μl water, 1.5 μl forward and 0.3 μl reverse primers (0.2 μM final concentration), and 1.5 μl DNA. A negative control was also included into which water was added in the place of DNA. A portion of each reaction was electrophoresed in a 0.8% agarose gel and stained with GelGreen (Phenix, Candler, NC). The DNA band for each sample was visually categorized by brightness and size for quality control. Samples were pooled (5 μl of each reaction for samples with bright bands, 10 μl for faint samples with bands, and 12 μl for samples with non-visible bands) and purified with the QIAquick PCR Purification Kit (QIAGEN, Valencia, CA) according to the manufacturer’s instructions. Pooled, purified amplicons were sequenced at the University of California-Davis DNA Technologies Core Facility on an Illumina MiSeq sequencing platform.

#### Sequence Analysis

The QIIME software package (version 1.8) was used to analyze the results of the Illumina sequencing run. llumina V4 16S rRNA gene sequences were demultiplexed and quality filtered with default settings unless otherwise specified^35^. Reads were truncated after a maximum number of 3 consecutive low quality scores. The minimum number of consecutive high quality base calls to include a read (per single end read) as a fraction of the input read length was 0.75. The minimum acceptable Phred quality score was set at 20. Similar sequences were clustered into operational taxonomic units (OTUs) using open reference OTU picking with UCLUST software^36^. Taxonomy was assigned to each OTU with the Ribosomal Database Project (RDP) classifier^37^ and the RDP taxonomic nomenclature^38^. OTU representatives were aligned against the Greengenes core set^39^ with PyNAST software^40^. The alignment was filtered with the filter_alignment.py script to remove positions which are gaps in every sequence and a phylogeny was constructed with FastTree^41^. Beta diversity analysis was performed using a weighted UniFrac distance matrix^42^. The OTU table was normalized by cumulative sum scaling and the adonis function with 999 permutations was used to detect differences in the community structure (as a whole) by sample category^43^,^44^. OTUs were then merged at the genus level, *Bifidobacterium* reads apportioned into (sub) species as measured by Bif-TRFLP/BLIR (see below) and metagenomeSeq used to detect differential taxa abundance between sample classes, with FDR correction^44^.

### Community State Analysis

After taxa were aggregated at the species level, microbiomes were clustered using the Bray-Curtis distance metric and a Non-metric multidimensional scaling (NMDS) ordination method. The Bray-Curtis metric was chosen because it does not account for phylogenetic relatedness and therefore provides a better separation of communities that are dominated by closely related species, as occurs with infant gut microbiomes. Based on a screeplot and eigenvalues, the first 6 dimensions were retained for the NMDS ordination. The number of clusters (K = 5) was determined using the gap statistic. The members of the five clusters were determined using the partition around medoids algorithm, pam, in R.

### Bif-TRFLP (terminal restriction fragment length polymorphism)

Because short amplicon-based sequencing often lacks the ability to resolve species-level taxonomic distinctions^45^, the method of Lewis *et al*. was used to perform the *Bifidobacterium*-specific terminal restriction fragment length polymorphism (Bif-TRFLP) assay to clarify the composition of bifidobacteria in infants^46^. Briefly, DNA from feces was amplified in triplicate by PCR using primers NBIF389 (5′-[HEX]-GCCTTCGGGTTGTAAAC) and NBIF1018 REV (5′-GACCATGCACCACCTGTG) (Table S1). DNA was purified using the Qiagen Qiaquick PCR purification kit (QIAGEN, Valencia, CA) and then cut with restriction enzymes AluI and HaeIII. The resulting fragments were analyzed on an ABI 3100 Capillary Electrophoresis Genetic Analyzer at the UC Davis College of Biological Sciences Sequencing Facility. The data was analyzed with PeakScanner 2.0 software (Applied Biosystems, Carlsbad, CA) and sizes were compared against the published database for species identification.

### Bifidobacterium Longum-Infantis Ratio (BLIR)

A PCR-based assay, BLIR, was used to determine which subspecies of *B. longum* were present in each sample and to gain an estimate of their abundance relative to each other^47^. Three primers (FWD_BL_BI (5′-[HEX]-AAAACGTCCATCCATCACA), REV_BL (5′-ACGACCAGGTTCCACTTGAT), and REV_BI (5′-CGCCTCAGTTCTTTAATGT)) were used to amplify the targeted genomic regions. FWD_BL_BI is complementary to a sequence in both subspecies while REV_BL and REV_BI are complementary to nearby sequences in only *B. longum* subsp. *longum* and *B. longum* subsp. *infantis*, respectively. FWD_BL_BI and REV_BL amplify a fragment of the *B. longum* spp. *longum* genome 145 bp in length, while FWD_BL_BI amplify a fragment of the *B. longum* subsp. *infantis* genome 114 bp in length.

DNA from each sample was amplified by PCR using 0.5 μl of 10 μM stock of each of the above primers, 12.5 μl GoTaq Green Master Mix (Promega), 1 μl of 25 mM MgCl_2,_ 1 μl of template DNA, and 9 μl of nuclease free water. Cycling conditions were 95 **°**C for 2 minutes, 30 cycles of 95 °C for 1 minute, 54 **°**C for 1 minute, and 72 **°**C for 30 seconds, followed by a 72 **°**C extension for 5 minutes. PCR products were purified from the mixture using the QIAquick PCR purification kit (Qiagen) and diluted 1:10. 1.5 μl of the dilutions were analyzed by capillary electrophoresis on an ABI 3100 Capillary Electrophoresis Genetic Analyzer at the UC Davis College of Biological Sciences Sequencing Facility. The HEX fluorophore on the common primer allowed detection and differentiation of amplicon sizes and a rough quantitation of the abundance of each amplicon based on peak area when the samples were analyzed with PeakScanner 2.0 software (Applied Biosystems, Carlsbad, CA). A positive control was included with each PCR run to ensure potential amplification of both *B. longum* subsp. *longum* and *B. longum* subsp. *infantis* products.

### Determination of Secretor Genotype

DNA from saliva was extracted using the Qiagen Blood & Tissue Kit and protocol (QIAGEN, Valencia, CA). Secretor genotype was determined as in Lewis *et al*.^31^. Briefly, genomic DNA was amplified with primers FUT2-F (5′-CCTGGCAGAACTACCACCTG) and FUT2-R (5′-GGCTGCCTCTGGCTTAAAGA) (Georgia- TECHNE TC-5000 thermocycler, Armenia- BOECO THERMAL CYCLER TC-PRO), which produces a 608 bp amplicon. Samples with low amplicon concentrations were reamplified with 35 cycles of PCR for all samples, and 50 for some difficult samples. Negative controls were run with each set of PCRs, and results were not used unless the controls were free of amplification in the expected size range. The individual performing the PCRs’ genotype was non-secretor, which should limit false secretor designations due to lab worker contamination.

The amplicons were then digested with BfaI, which cuts the DNA of individuals containing the mutated non-secretor rs601338 SNP (G → A, Trp → Ter) allele of the FUT2 gene, the predominant non-secretor mutation in Caucasians. The resulting digests were electrophoresed on a 2% agarose gel for ~2 hours at 80 V and the resulting bands were visualized using GelGreen dye under UV light. Individuals possessing a secretor allele produce a 608 bp band on the gel after digestion, while a non-secretor allele produces two bands with sizes of 516 bp and 92 bp. In this way it is possible to easily distinguish both homozygote genotypes from each other and from heterozygotes. Heterozygotes were counted as secretors for statistical analysis, therefore contamination by non-secretor DNA would not change the designation of individuals for this purpose.

## Results

### Cohort Metadata

In total, 205 samples were collected, 133 from Armenia, and 72 from Georgia. Mother’s saliva (for secretor genotyping) was unavailable for 31 of the 53 Armenian infants, and 11 of the 28 Georgian infants. Four samples (38b, 48.a, 48.b, 48.c) were found to be duplicates and excluded from analysis. A full description of the metadata of the two cohorts is found in Table 1, including classification by country, mother’s secretor status, and day of sample collection.

### General Microbiome Trends

Four hundred and twenty six genus-level taxa were identified in the sequencing data. Table 2 lists all taxa with a minimum average relative abundance of 0.5%, and breaks down the bifidobacteria by species according to the Bif-TRFLP/BLIR data. Bifidobacteria were the dominant taxa in the infant gut microbiota in these infants, comprising 44% of all reads. The most abundant bifidobacterial species was *B. breve*, which accounted for 16.3% of the total microbiota.

**Figure 1:**
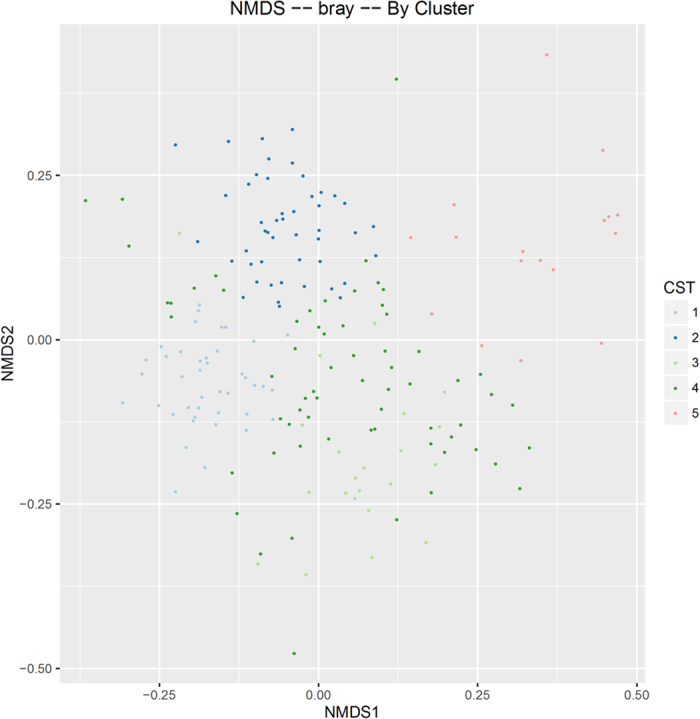
Infant gut microbiome clusters using a Bray-Curtis distance metric and a Non-metric multidimensional scaling (NMDS) ordination. CST = community state type.

**Figure 2:**
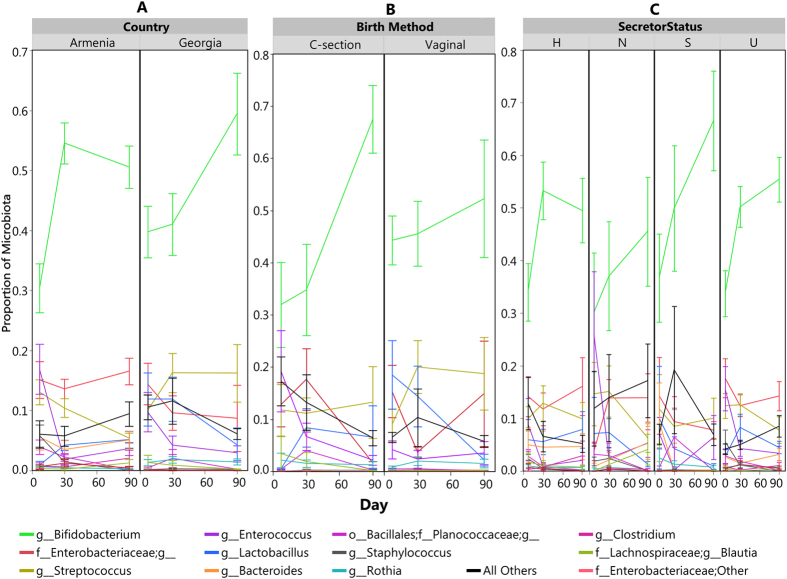
Members of the infant gut microbiome with greater than 0.05% average relative abundance over time based on proportion of sequencing reads. Error bars are standard error. (**A**) Average proportion of major gut microbes by country. (**B)** Average proportion of major gut microbes by birth method. (**C)** Average proportion of major gut microbes by mother’s secretor status. H = heterozygote, S = homozygote secretor, N = non-secretor, U = unknown.

**Figure 3:**
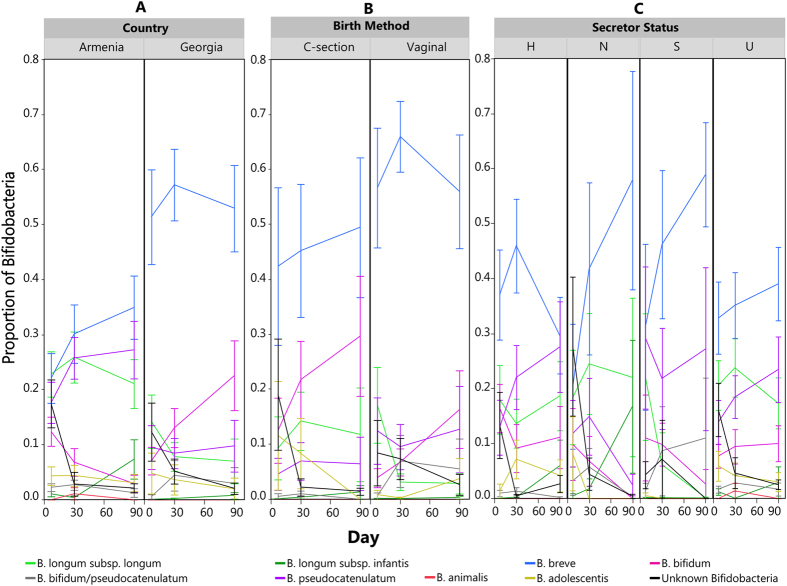
Average relative abundance of each member within the genus *Bifidobacterium* over time, based on the Bif-TRFLP/BLIR data. Error bars are standard error. (**A)** Average proportion of bifidobacteria by country. (**B)** Average proportion of bifidobacteria by birth method. (**C)** Average proportion of bifidobacteria by mother’s secretor status. H = heterozygote, S = homozygote secretor, N = non-secretor, U = unknown.

A community state analysis was conducted on the sequencing-based data to explore microbial community structure (see Methods). This unsupervised exploration of the data yielded a set of 5 community state types (Fig. 1): a community dominated by an OTU assigned to the *B. longum* group (Figures S1, S6), a community dominated by an OTU assigned to *B. adolescentis* (Figures S2, S7), a community dominated by *Bifidobacterium* of unresolved species (Figures S3, S8), a mixed community low in *Bifidobacterium* (Figures S4, S9), and an *Enterococcus*-dominant community (Figures S5, S10). The biggest driver of community state seems to be whether or not the community is dominated by bifidobacteria. The samples associated with the *Enterococcus*-dominant group were mostly from Day 7. Also, none of the samples in this group came from babies with homozygous secretor mothers. To avoid overemphasizing the importance of minor community members, the analysis of differentially abundant taxa was confined to only bifidobacteria (at a species level determined by the Bif-TRFLP and BLIR methods) and other major genera. Figure 2 shows a breakdown of the levels of these taxa over time by various metadata categories and Fig. 3 shows the breakdown of bifidobacterial species in the same way.

Individual infants were found to be similar to themselves over time, with an Adonis R^2^ value of 0.027 for subject number, indicating that individuality was responsible for 2.7% of the variation in the microbiota (Pr(>F) = 0.001). To investigate the pattern of bacterial colonization of the gastrointestinal tract over time, the composition of the infant microbiota was compared at each time point. Time was found to be a significant influence on the infant microbiota (Pr(>F) = 0.001) and was responsible for 2.3% of the variation in the microbiota (Adonis R^2^ of 0.023). Between days 7 and 90, *B. breve, B. longum* subsp. *infantis,* and lactobacilli increased in abundance (p = 0.004, 0.039, and 9.29 × 10^−5^ respectively), while staphylococci, and the Planococcaceae family decreased in abundance (p = 3.18 × 10^−8^ and 4.86 × 10^−5^ respectively). In total, 30 of the 426 genera were differentially abundant between the earliest time point and the latest. Table 3 contains a list of the major taxa enriched in each time point and metadata group, while Supplementary Table S1 contains a complete list of taxa differentially abundant between each pair of time points and other metadata categories.

### Comparison Between Countries

The overall composition of the microbiota of the infant gastrointestinal tract was tested for differences between infants in the two countries. Figure 2A shows a summary of the major constituents of the infant gut bacterial community of the two countries (average abundance cutoff of 0.5%), and how they shift over the first 90 days of life. The overall patterns of colonization were similar between the two countries, as bifidobacteria dominated the infant fecal microbiome of both countries, with significant amounts of enterobacteria, streptococci, enterococci, lactobacilli, *Bacteroides*, and staphylococci also present. Country of origin accounted for 8.4% of total variation between samples (Adonis R^2^ value of 0.084, (Pr(>F) = 0.001)). Of the 426 total genera, 196 were found to be differentially abundant between the two countries. There were statistically significant differences (p < 0.05) found between the major community members of the two countries. In general infants in Armenia had more Enterobacteriaceae, Planococcaceae, *Streptococcus, Enterococcus, Bacteroides, Clostridium,* and *Staphylococcus. B. breve* was the most abundant bifidobacterial species in both countries. Of the bifidobacteria, Armenian infants had more *B. pseudocatenulatum* and *B. longum* subsp. *longum*. There were no bifidobacterial taxa more abundant in Georgian infants that reached statistical significance. Figure 3A shows the results of the Bif-TRFLP/BLIR analysis by country.

### C-section vs. Vaginal Birth

In addition to the other metadata collected, birth method was tracked and a subset of 11 of the 28 infants from the Georgian cohort were born by Caesarian section. We explored the impact of this first exposure to microbes on the gut microbiota of the infants. Figure 2B shows a summary of the major constituents of the infant gut bacterial community of infants born by both methods, and Fig. 3B shows the bifidobacterial species-level data. Bifidobacteria, for example, dominated the communities of both types of infants. About 3.1% of the variation in the overall community structure was attributable to birth method (Pr(>F) = 0.001). Several major community members (average abundance cutoff of 0.5%) were generally different between infants born by C-section and vaginally. Vaginally born infants were higher in *B. pseudocatenulatum* (p = 0.03), Enterobacteriacaea; g_(p = 0.01), *Streptococcus* (p = 0.008)*, Staphylococcus* (p = 0.005), and Planococcaceae; g_(p = 0.0007). Due to the lack of C-section born infants in Armenia, however, it is difficult to fully disentangle the effect of country from that of birth method.

### Secretor Status Differences

We also wished to investigate the effect of the mother’s secretor status on the infant microbiota. We were able to determine the secretor genotype for 22/53 (41.5%) Armenian mothers and 17/28 (60.7%) of Georgian mothers. A breakdown of the overall infant gut bacterial community data by mother’s genotype in found in Fig. 2C. We grouped the infants samples into 4 subsets depending on the mother’s secretor genotype, homozygote secretors (S), homozygote non-secretors (N), heterozygotes (H), and “unknown” mothers for which salivary DNA was not available (U). Bifidobacteria were the dominant member of the microbiota in all four groups, and their abundance generally increased over time (except for the H group from day 30 to 90, where there was a small decrease) (Fig. 2C). Approximately 1.6% of the variation in the microbial community was attributable to mother’s secretor status, though the effect was only marginally significant (Pr(>F) = 0.066, mothers of known secretor status classes only with heterozygotes are counted as secretors).

**Figure 4:**
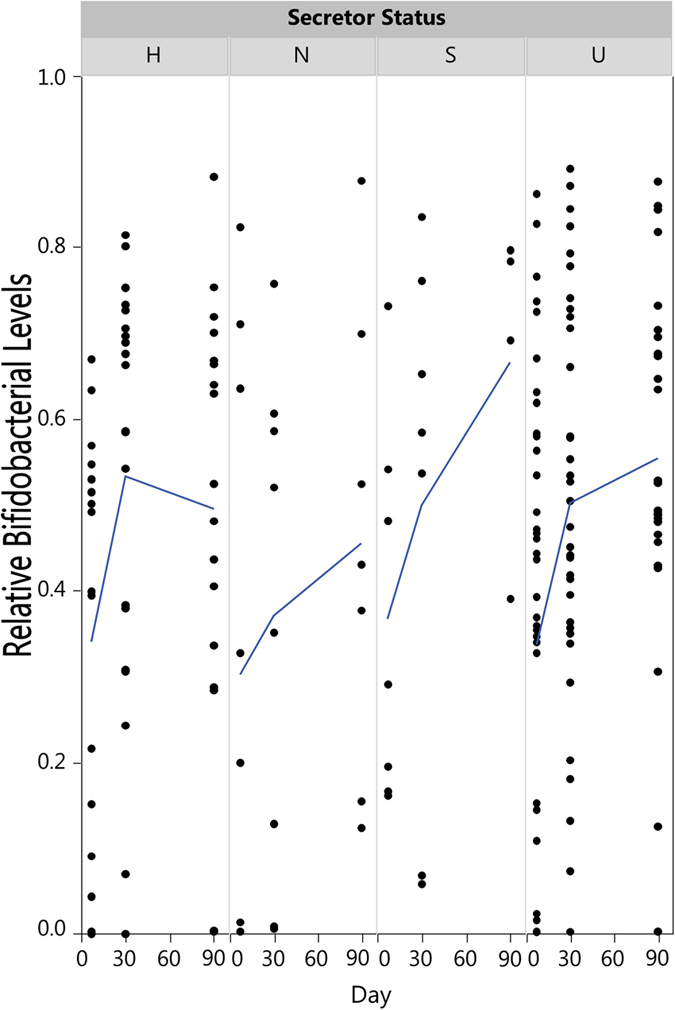
Bifidobacterial relative abundance over time by secretor genotype. The lines show the average bifidobacterial abundance of each group over time. H = heterozygote, S = homozygote secretor, N = non-secretor, U = unknown.

However, a trend was observed where the average amount of bifidobacteria increased more quickly in secretor-fed infants than non-secretor-fed infants while heterozygote-fed and unknown-fed infants contained intermediate levels of bifidobacteria by day 90. This trend appears to be driven in part by fewer infants having near-zero levels of bifidobacteria in the secretor-fed infants (Fig. 4). There were no differentially abundant major taxa between infants fed by mothers of known secretor status (heterozygotes included as secretors) and infants fed by know non-secretor mothers. At the level of bifidobacterial species, *B. breve* was the present at the highest level across all infants (Fig. 3C).

## Discussion

The composition of the gut microbiota is thought to impact infant health in numerous ways. The establishment of the gut microbiota can influence an individual’s lifelong health^48^,^49^. In the first years of life the infant gut microbiome progresses through age-associated compositional changes^50^. The infant gut microbiota is sensitive to disruption early in life, as it has unfilled niches and has not developed mature levels of colonization resistance^5^,^51^,^52^,^53^. From an evolutionary perspective, alterations in gene content or expression that assist an infant in fostering select, beneficial, microbial communities would be advantageous. For example, infants appear to suppress their immune system early in life to aid in shaping their microbiota^54^.

Maternal genes influencing the infant gut microbiota may be under selection as well, as long as the maternal investment benefits the infant sufficiently to overcome the inherent parent-offspring conflict over resources^55^. For example, mother’s milk has been shown to affect the infant gut microbiota in numerous ways^7^. Moreover, exposure to microbes via birth method (C-section vs. Vaginal birth) and other behavioral or circumstantial factors may also play a role in the development of the microbiota independent of the selective environmental conditions within the gut. Due to the observed differences in the microbiota of infants from different countries (Norway^26^, Sweden^27^, Canada^28^, Italy^29^, Switzerland^30^, Bangladesh^22^, the USA^31^, Malawi and Finland^32^) we sought to investigate and disentangle community differences caused by selection (i.e. maternal secretor status) from differences caused by environmental exposure (regionality and birth method).

Armenia and Georgia are neighboring countries, but possess distinct languages and cultural differences^56^. Country of birth was found to have the highest Adonis R^2^ value of any factor tested, indicating that regionality has a relatively large impact on the microbial community. Lacking both a thorough ethnography focused on behavioral differences between the two groups that may influence the exposure to colonizing microbes and relevant data on the environmental pressures experienced by the microbes in the gut, the mechanisms which drive the differences in the infant microbiota of the two countries are not identifiable. It is however striking to observe the differences in infants from cities which, while in two different countries, are only approximately 175 km in distance from each other. Despite these differences, bifidobacteria dominated the community of infants in both countries, and *B. breve*, a species of bifidobacteria common to infants, was the found in the highest levels in both cohorts. Similar to other reports on the infant microbiome, the major non-bifidobacterial taxa present in the infants in our study were enterobacteria, streptococci, enterococci, lactobacilli, *Bacteroides, Clostridium,* and staphylococci^22^,^27^,^28^,^29^,^30^,^31^,^57^. While this study is not focused on the minor taxa in these infants, there were many differences in the lower abundance taxa between the two countries, perhaps indicating differential exposure to allochthonous environmental microbes.

C-section birth has been associated with altered community assembly in infants, often thought to be the result of differential exposure to microbes from vaginally-born infants. Currently, over 30% of babies are born by Cesarean section in the United States, and approximately 82% of cases of methicillin-resistant *Staphylococcus aureus* infections occur in this subset of infants^58^. In other countries, C-sections are known to reduce the rate of bifidobacterial colonization^59^, but that trend was not observed in this and other studies^22^. We also failed to observe early enrichment of lactobacilli in the vaginally-born infants, a commonly observed phenomenon ascribed to the transfer of vaginal lactobacilli to the infant^60^.

Apart from the effect of microbial exposure, many studies suggest that the consumption of glycans found in milk influences the initial microbial colonization of the neonatal gastrointestinal tract^10^,^31^,^61^,^62^. Specifically, the glycan content in milk may shape the microbiome through its selective fermentability by members of the microbial community^25^,^31^,^61^,^63^,^64^. In this study the major bifidobacterial species present was *B. breve,* a common infant commensal known to consume human some milk oligosaccharides^65^,^66^,^67^. Indeed, *B. breve* and *B. longum* subsp. *infantis*, two species known to consume HMOs^65^,^68^, were both found to be enriched over the course of extended breastfeeding, supporting the hypothesis that the ability to consume these carbon sources is an adaptive trait for infant microbes. Early establishment of bifidobacteria is thought to be beneficial in numerous ways. High levels of bifidobacteria were shown to be predictive of better immune response to vaccines^22^. Other benefits associated with higher bifidobacteria include better development of an infant’s immature immune system and protection from colonization by pathogens^17^,^22^,^69^,^70^.

Secretor status in general is known to affect the gut microbiota of both adults and infants^31^,^71^,^72^. Secretor status also alters both the bifidobacterial species profiles and absolute levels of bifidobacteria in the gastrointestinal tract of adults and infants, where secretors have higher bifidobacterial abundance^31^,^73^,^74^. Maternal secretor status has been shown to impact infant resistance to infection by enteric pathogens, including members of the Proteobacteria phylum^75^,^76^. Maternal secretor status has also been shown to impact the types and amounts of bifidobacteria in the infant^31^. The infant’s own glycosylation system is also a potential influence on the development of the gut microbiota early in life, however the temporal variation of an infant’s glcosyltransferase expression, including the FUT2 gene, remains to be elucidated. While they do not reach statistical significance, the trends reported here support previous data that suggest that secretor mothers promote bifidobacteria establishment more strongly than non-secretor mothers. The failure to reach statistical significance, may possibly be due in part to the low number of subjects for which we had secretor status data. We were also unable to confirm our previous results correlating the abundance of *B. longum* subsp. *infantis* with the mother’s secretor status in this cohort, likely due to the low amount of *B. longum* subsp. *infantis* observed in these infants (n = 6 for infants with *B. longum* subsp. *infantis* levels of over 1%). We also did not observe co-enrichment of *B. breve* and *B. bifidum* abundances that would be indicative of previously hypothesized *in vivo* cross-feeding between the two species, even in infants fed by mothers of known secretor status^77^,^78^. The low percentages of variation explained by the results of the Adonis tests indicate that the sample metadata recorded this study may not fully capture the suite of factors that explain the majority of the differences between sample classes. Indeed, the unsupervised clustering of the data demonstrated that there were few relationships between the sample metadata and the resulting community state types, suggesting that microbial community structure in breastfed babies transcends geography, mode of delivery, and secretor status. The Enterococcus-rich communities were mostly from the earliest time point, implying that nearly all breastfed babies later reach the Bifidobacteria-dominant state.

In summary, this study adds valuable information about the gut microbiome of previously unstudied populations. Breast-fed infants in Georgia and Armenia appear to follow well-established patterns of microbial colonization^5^. Country of birth was found be the largest influence on microbial community structure of the factors tested, signaling differences in the infant microbiota between the two closely-situated countries and raising the question of the degree to which the developing intestinal microbiota might be influenced by national boundaries and the cultural and genetic differences they represent. Previous findings regarding the influence of maternal secretor status of infant gastrointestinal microbiota colonization patterns were supported by our results here, including a non-significant trend of enriched bifidobacteria in secretor-fed infants. These findings may be useful as guidance in the future application of pre- and probiotic treatments for vulnerable infants in the new era of easier genetic testing and personalized medicine.

## Additional Information

**How to cite this article**: Lewis, Z. T. *et al*. The Fecal Microbial Community of Breast-fed Infants from Armenia and Georgia. *Sci. Rep.* **7**, 40932; doi: 10.1038/srep40932 (2017).

**Publisher's note:** Springer Nature remains neutral with regard to jurisdictional claims in published maps and institutional affiliations.

## Supplementary Material

Supplementary Information

Supplementary Table 1

## Figures and Tables

**Table 1:** Cohort metadata.

	Country							
	Armenia				Georgia			
	Day				Day			
Secretor Status	7	30	90	All	7	30	90	All
H (Heterozygote)	11	12	10	33	9	8	7	24
N (Homozygote non-secretor)	7	6	6	19	3	3	2	8
S (Homozygote secretor)	2	2	1	5	5	6	3	14
U (Unknown)	30	25	21	76	11	11	4	26
All	50	45	38	133	28	28	16	72

Breakdown of samples by time point, secretor status, and country.

H = heterozygote, S = homozygote secretor, N = non-secretor, U = unknown.

**Table 2:** Summary of all taxa with a minimum average relative abundance of 0.5%, and breakdown of the bifidobacteria by species according to the Bif-TRFLP/BLIR data.

Taxa	Average Abundance (%)
g_*Bifidobacterium*; s_All	44.0
s*_breve*	16.3
s*_longum* subsp. *longum*	8.3
s*_pseudocatenulatum*	8.1
s*_bifidum*	4.2
s*_*Unknown	3.5
s*_adolescentis*	1.7
s*_bifidum/pseudocatenulatum*	1.0
s*_longum* subsp. *infantis*	0.8
s*_animalis*	0.1
f_Enterobacteriaceae; g_	13.5
g_*Streptococcus*	11.6
g_*Enterococcus*	7.6
g_*Lactobacillus*	5.6
g_*Bacteroides*	3.1
f_Planococcaceae; g_	1.8
g_*Staphylococcus*	1.8
g_*Rothia*	0.8
g_*Clostridium*	0.7
g_*Blautia*	0.7
f_Enterobacteriaceae; Other	0.5
Total	91.9

**Table 3:** List of the major taxa and bifidobacterial species enriched (p < 0.05) in each time point and metadata group.

Major Taxon	Country Comparison			Birth Method Comparison			Day 7 vs. Day 30			Day 7 vs. Day 90			Day 30 vs. Day 90		
	Country	Adjusted p value	β coefficient	Birth Method	Adjusted p value	β coefficient	Day	Adjusted p value	β coefficient	Day	Adjusted p value	β coefficient	Day	Adjusted p value	β coefficient
g_Bifidobacterium															
s_breve							Day 7	0.046	−2.60	Day 90	0.004	3.27			
s_longum subsp. longum	Georgia	0.012	−2.27												
s_pseudocatenulatum	Georgia	0.0004	−3.33	Vaginal	0.031	3.38									
s_bifidum															
s_other							Day 7	0.046	2.12						
s_adolescentis															
s_bifidum/ pseudocatenulatum															
s_longum subsp. infantis										Day 90	0.039	1.60			
s_animalis															
f_Enterobacteriaceae;g_	Georgia	1.95E-06	−2.44	Vaginal	0.012	2.10									
g_Streptococcus	Georgia	0.0001	−1.59	Vaginal	0.008	1.73									
g_Enterococcus	Georgia	0.022	−1.37				Day 7	0.021	2.02						
g_Lactobacillus							Day 7	5.31E-05	−2.78	Day 90	9.29E-05	2.50			
g_Bacteroides	Georgia	0.042	−1.21												
f_Planococcaceae;g_	Georgia	7.04E-11	−2.74	Vaginal	0.0007	2.29				Day 90	4.86E-05	−2.43	Day 90	0.002	−1.78
g_Staphylococcus	Georgia	2.86E-08	−2.86	Vaginal	0.005	2.40				Day 90	3.18E-08	−3.72	Day 90	4.43E-08	−2.78
g_Rothia															
g_Clostridium	Georgia	0.024	−1.04												
g_Blautia															
f_Enterobacteriaceae; Other	Georgia	8.65E-05	−2.10												

The “Country”, “Birth Method”, and “Day” columns indicate the base group for comparison, and the sign of the corresponding β coefficient indicates whether the taxa is enriched (+) or depleted (−) in the group specified in the column to its left. Non-significant results are left blank.
