# Supplementary material for: The Fecal Microbial Community of Breast-fed Infants from Armenia and Georgia
Source: Sci Rep. 2017 Feb 2;7:40932. doi: 10.1038/srep40932 (PMC5288704; doi:10.1038/srep40932)
Supplement: Supplementary Information [file srep40932-s1.pdf]

The Fecal Microbial Community of Breast-fed Infants from Armenia and Georgia.

Zachery T Lewis[1-3], Ketevan Sidamonidze[4], Vardan Tsaturyan[5], David Tsereteli[4], Nika Khachidze[4], Astghik Pepoyan[5], Ekaterine Zhgenti[4], Liana Tevzadze[4], Anahit Manvelyan[6], Marine Balayan[6], Paata Imnadze[4], Tamas Torok[7], Danielle G. Lemay[8], David A. Mills[1-3]*.

[1]Department of Food Science and Technology, University of California, Davis, CA

[2]Foods for Health Institute, University of California, Davis, CA

[3]Department of Viticulture and Enology, University of California, Davis, CA

[4]National Center for Disease Control and Public Health of Georgia, Tbilisi, Georgia

[5]IAHAHI (International Association for Human and Animals Health Improvement), Yerevan, Armenia

[6]Armenian National Agrarian University, Yerevan, Armenia

[7] Earth Sciences Division, Lawrence Berkeley National Laboratory, Berkeley, CA

[8]Genome Center, University of California, Davis, CA

***Corresponding Author**
Email: damills@ucdavis.edu
Office: 3142 RMI North Building
Phone: 530-754-7821
Fax: 530-752-0382
Address: University of California
One Shields Ave.
Davis, CA 95616-5270

Supplementary Information

Supplementary Figure S1: Community state type 1 labeled at the species level.

Supplementary Figure S2: Community state type 2 labeled at the species level.

Supplementary Figure S3: Community state type 3 labeled at the species level.

Supplementary Figure S4: Community state type 4 labeled at the species level.

Supplementary Figure S5: Community state type 5 labeled at the species level.

Supplementary Figure S6: Community state type 1 labeled at the genus level.

Supplementary Figure S7: Community state type 2 labeled at the genus level

Supplementary Figure S8: Community state type 3 labeled at the genus level

Supplementary Figure S9: Community state type 4 labeled at the genus level

Supplementary Figure S10: Community state type 5 labeled at the genus level

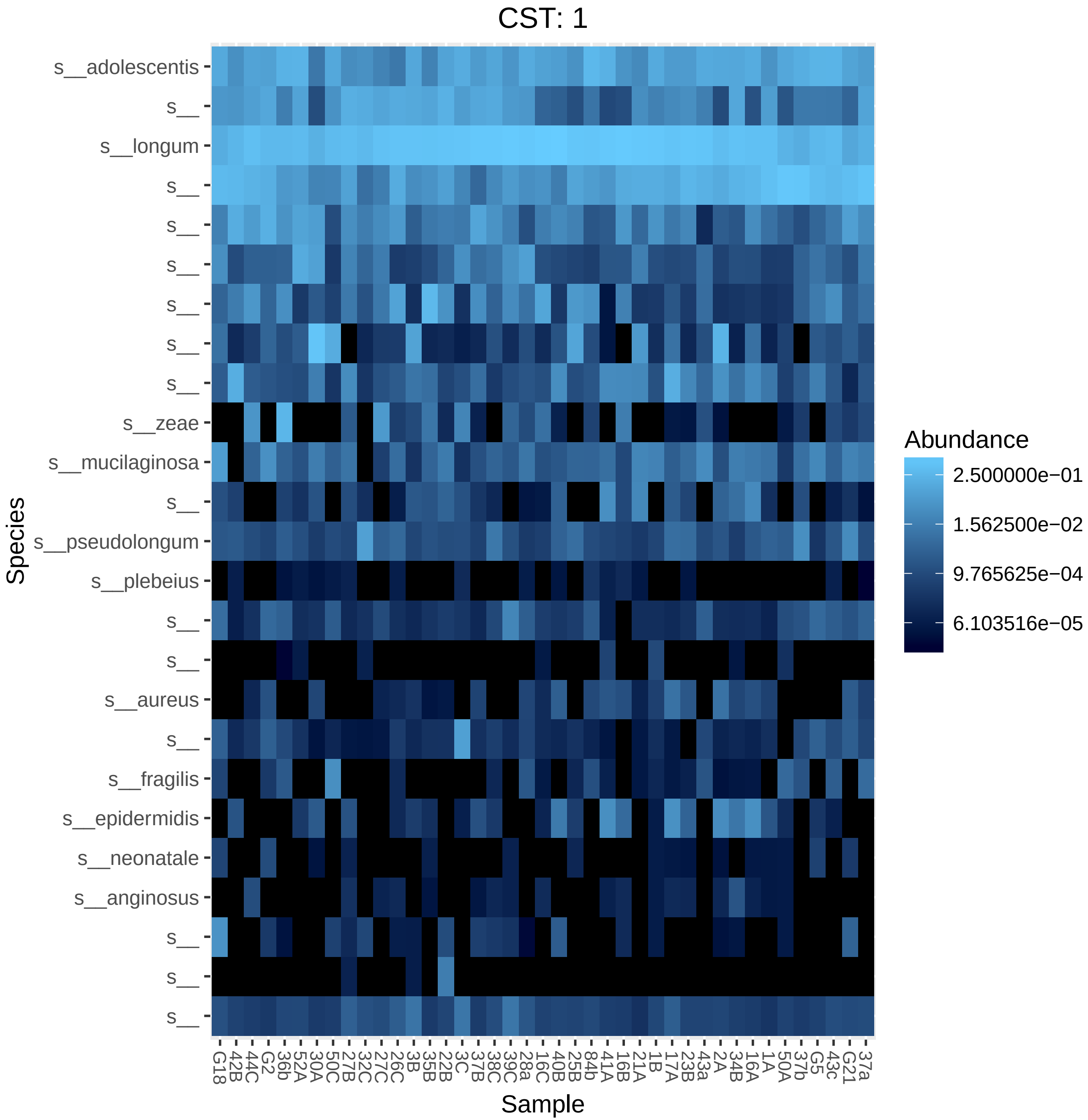

CST: 1
Species
s__adolescentis
s__
s__longum
s__
s__
s__
s__
s__
s__
s__zeae
s__mucilaginosa
s__
s__pseudolongum
s__plebeius
s__
s__
s__aureus
s__
s__fragilis
s__epidermidis
s__neonatale
s__anginosus
s__
s__
s__
Sample
G18 42B 44C G2 36b 52A 30A 50C 27B 32C 27C 26C 3B 35B 22B 3C 37B 38C 39C 28a 16C 40B 25B 84b 41A 16B 21A 1B 17A 23B 43a 2A 34B 16A 1A 50A 37b G5 43c G21 37a
Abundance
2.500000e−01
1.562500e−02
9.765625e−04
6.103516e−05

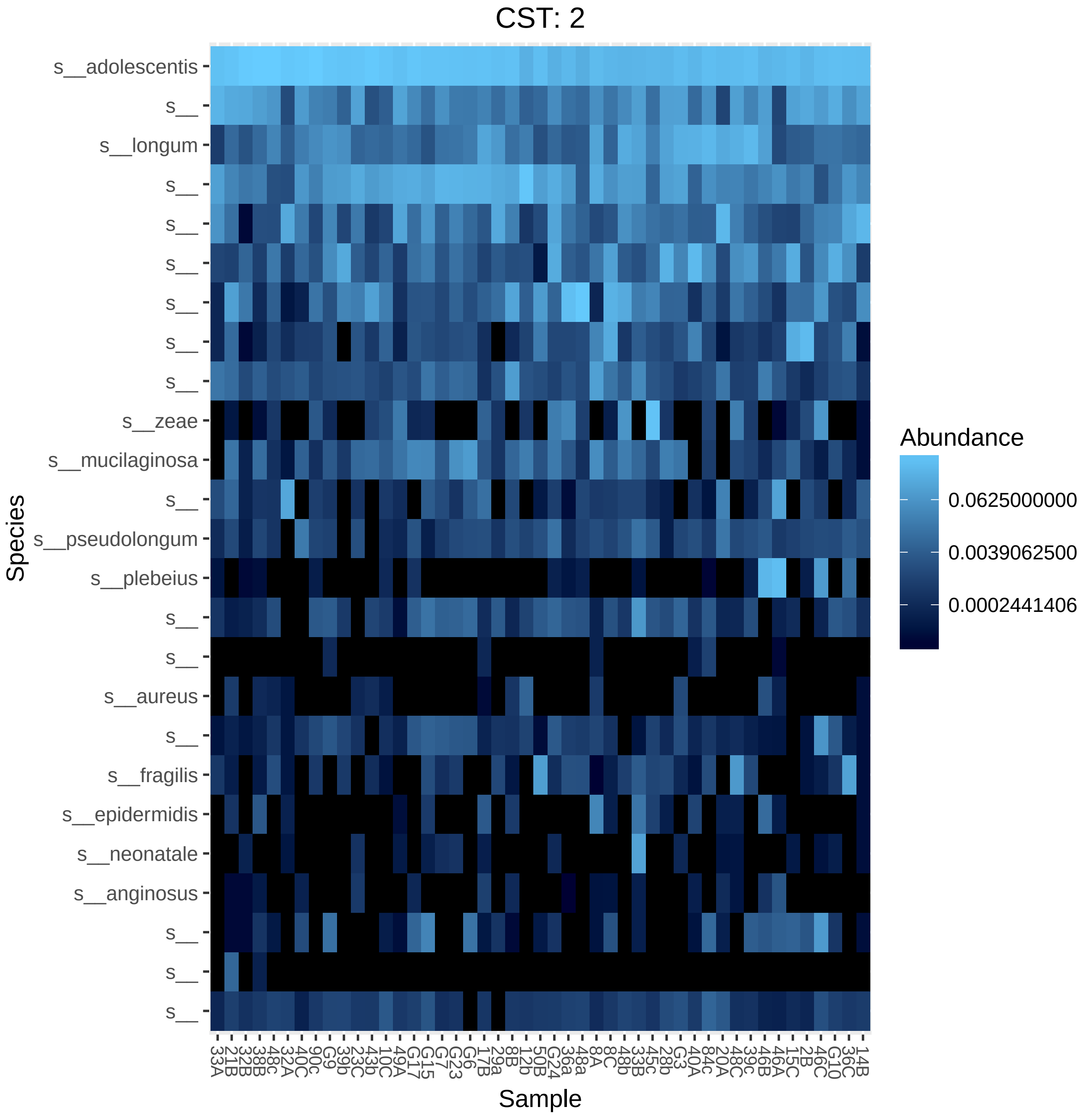
CST: 2
Species
s__adolescentis
s__
s__longum
s__
s__
s__
s__
s__
s__
s__zeae
s__mucilaginosa
s__
s__pseudolongum
s__plebeius
s__
s__
s__aureus
s__
s__fragilis
s__epidermidis
s__neonatale
s__anginosus
s__
s__
s__
Sample
33A 21B 32B 38B 48c 32A 40C 90c G9 39b 23C 43b 10C 49A G17 G15 G7 G23 G6 17B 29a 8B 12b 50B G24 36a 48a 8A 8C 48b 33B 45c 28b G3 40A 84c 20A 48C 39c 46B 46A 15C 2B 46C G10 36C 14B
Abundance
0.0625000000
0.0039062500
0.0002441406

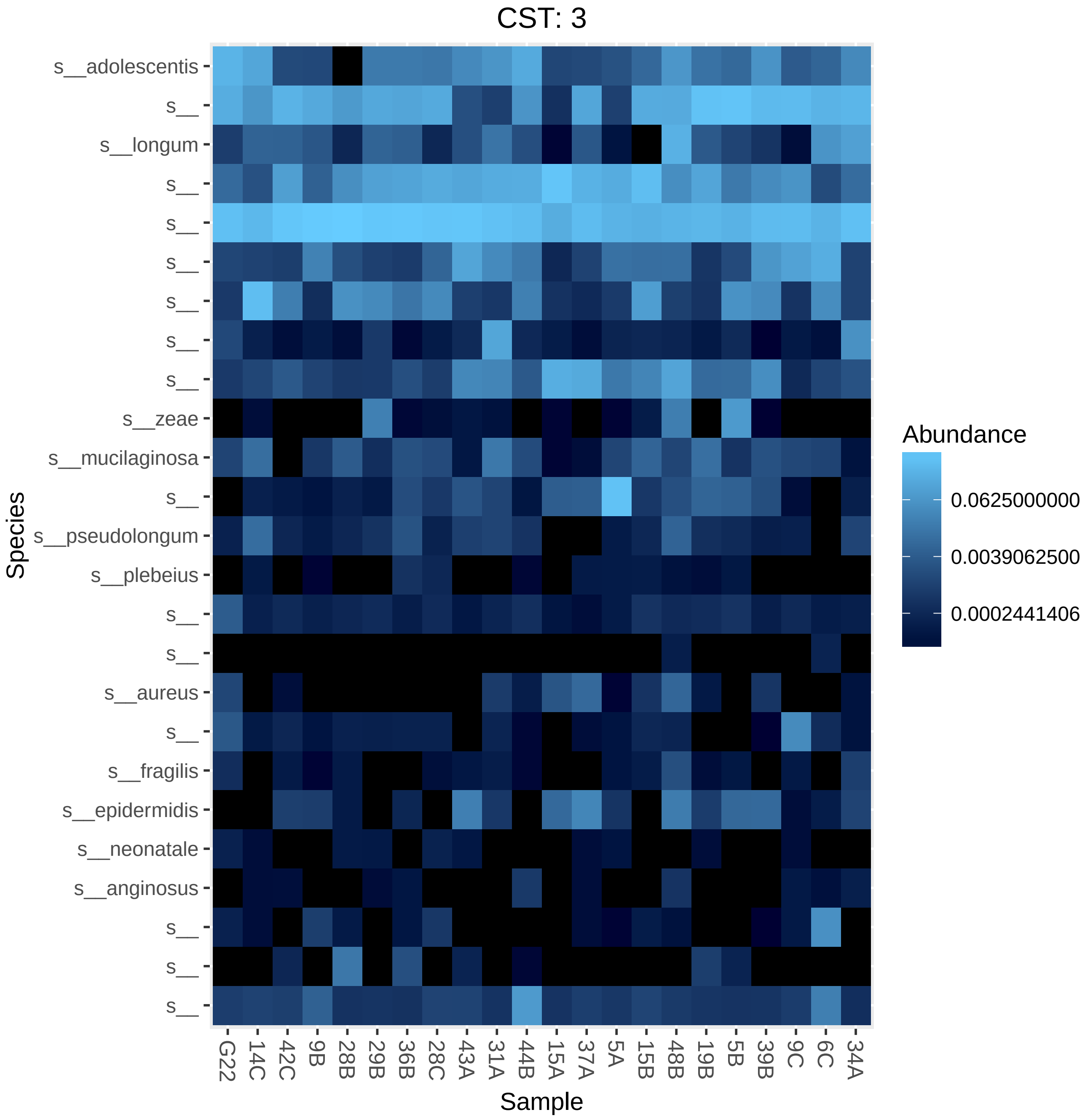

CST: 3
Species
s__adolescentis
s__
s__longum
s__zeae
s__mucilaginosa
s__pseudolongum
s__plebeius
s__aureus
s__fragilis
s__epidermidis
s__neonatale
s__anginosus
Sample
G22
14C
42C
9B
28B
29B
36B
28C
43A
31A
44B
15A
37A
5A
15B
48B
19B
5B
39B
9C
6C
34A
Abundance
0.0625000000
0.0039062500
0.0002441406

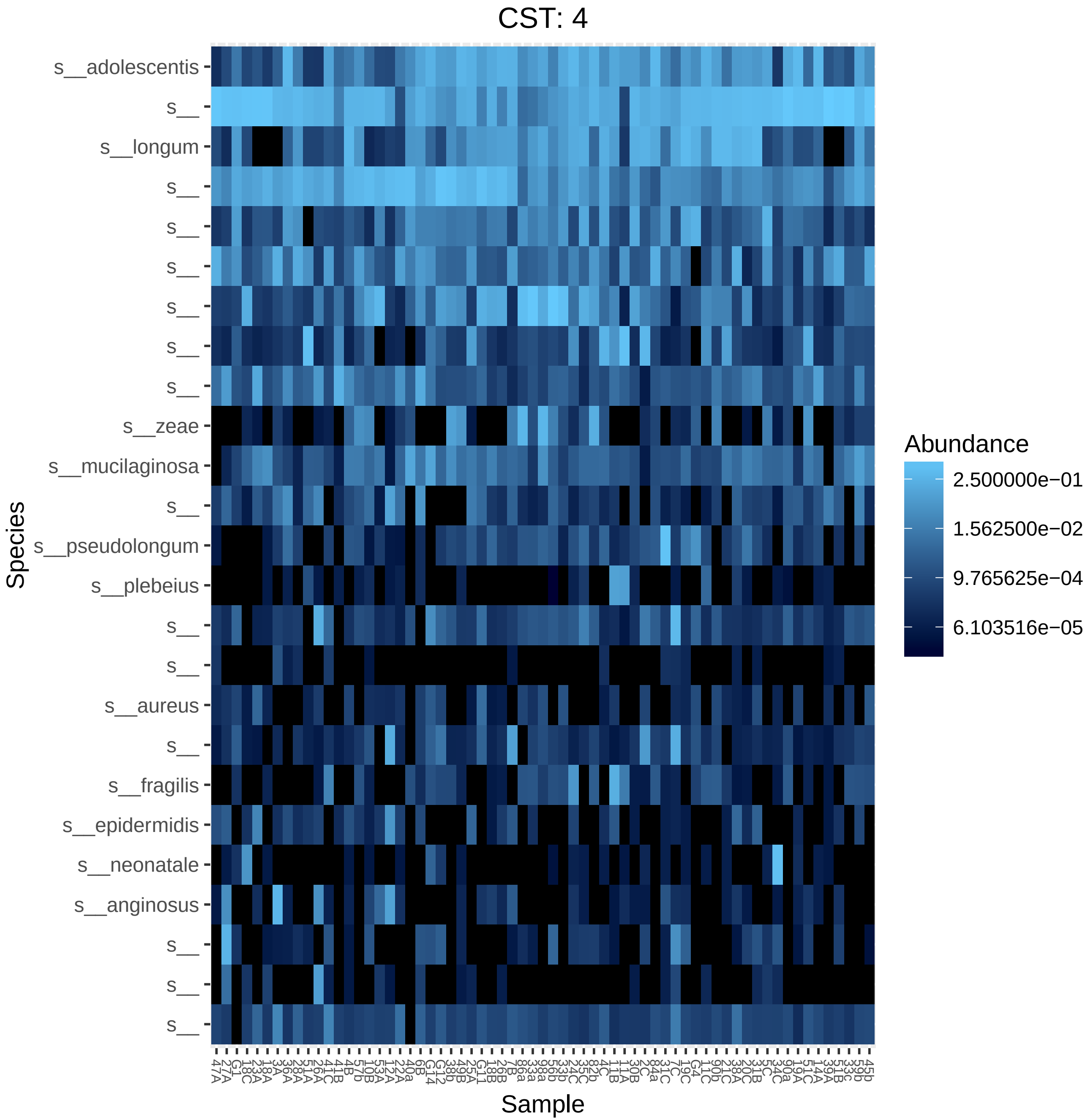
CST: 4
Species
s__adolescentis
s__
s__longum
s__
s__
s__
s__
s__
s__
s__zeae
s__mucilaginosa
s__
s__pseudolongum
s__plebeius
s__
s__
s__aureus
s__
s__fragilis
s__epidermidis
s__neonatale
s__anginosus
s__
s__
s__
Sample
47A 27A G1 18C 23A 18A 3A 36A 28A 51A 26A 41C 41B 4B 57b 10B 53A 12A 22A 40a 6B G14 G12 38b 49B 25A G11 18B 26B 7B 86a 83a 98a 56b 33b 24C 35C 82b 4C 11B 11A 30B 2C 84a 31C 7C 19C G4 11C 90b 21C 38A 20C 31B 5C 34C 90a 19A 51C 14A 39A 51B 33c 59b 45b
Abundance
2.500000e−01
1.562500e−02
9.765625e−04
6.103516e−05

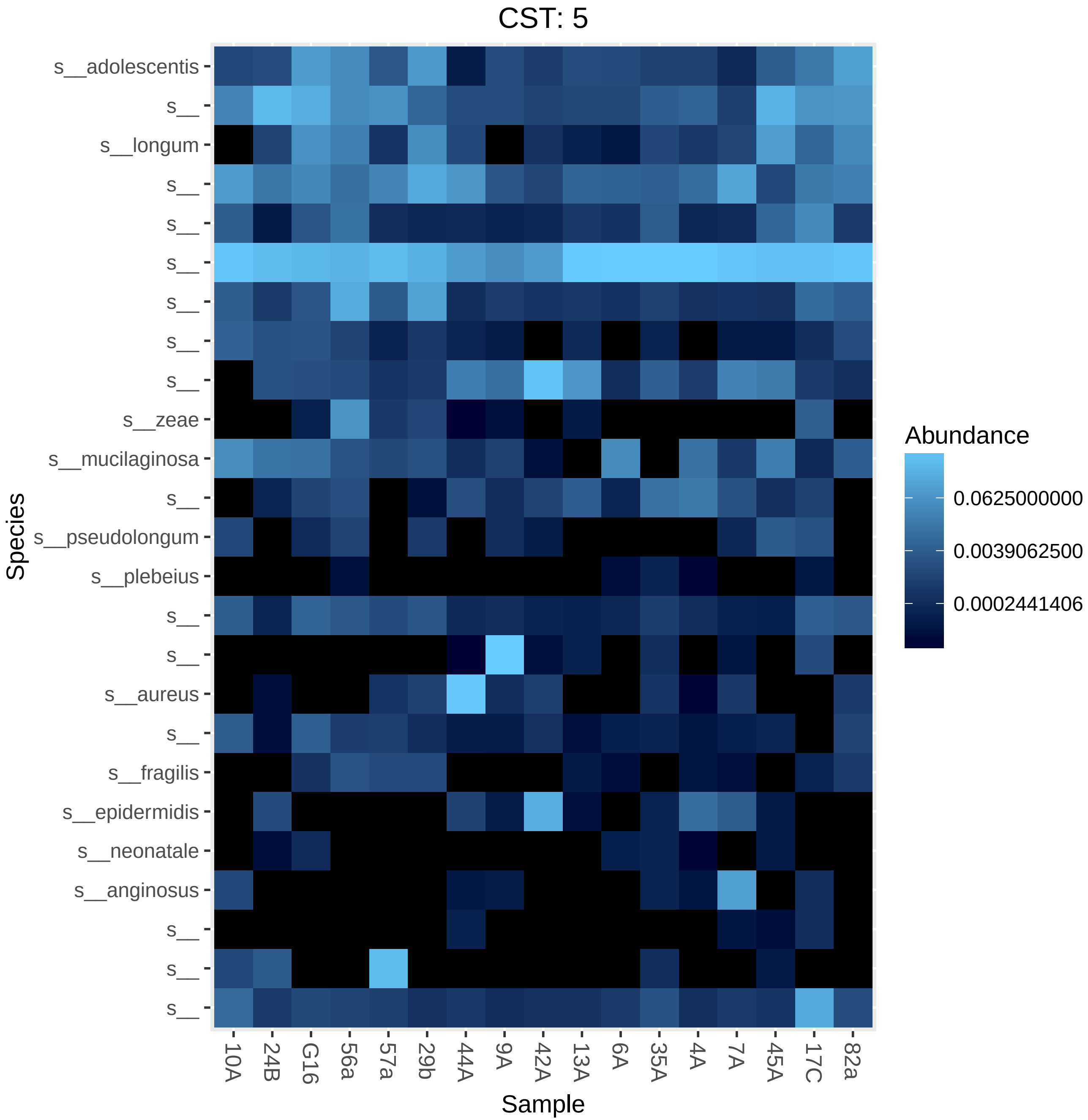
CST: 5
Species
s__adolescentis
s__
s__longum
s__
s__
s__
s__
s__
s__
s__zeae
s__mucilaginosa
s__
s__pseudolongum
s__plebeius
s__
s__
s__aureus
s__
s__fragilis
s__epidermidis
s__neonatale
s__anginosus
s__
s__
s__
Sample
10A
24B
G16
56a
57a
29b
44A
9A
42A
13A
6A
35A
4A
7A
45A
17C
82a
Abundance
0.0625000000
0.0039062500
0.0002441406

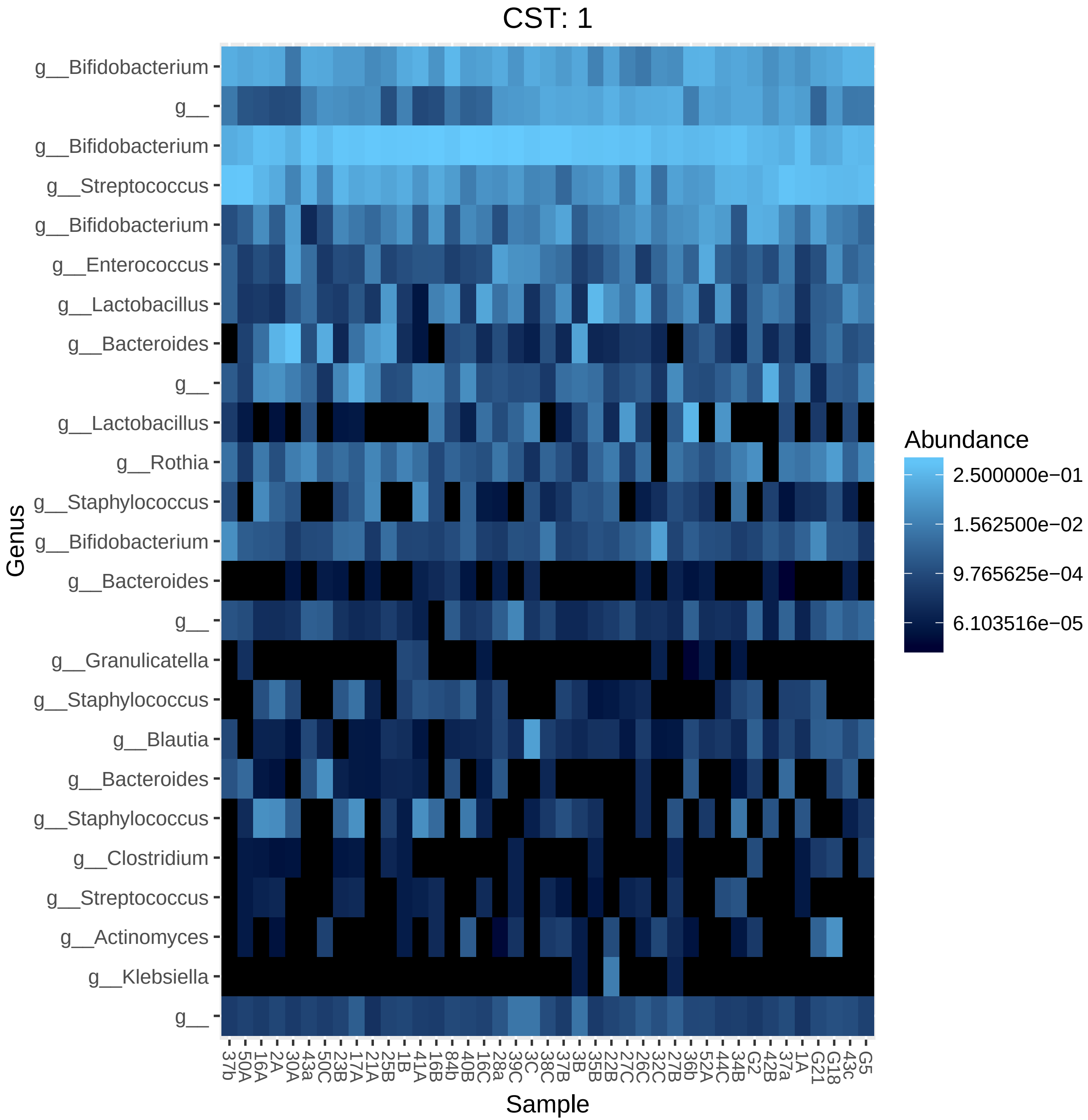

CST: 1
g__Bifidobacterium
g__
g__Bifidobacterium
g__Streptococcus
g__Bifidobacterium
g__Enterococcus
g__Lactobacillus
g__Bacteroides
g__
g__Lactobacillus
g__Rothia
g__Staphylococcus
g__Bifidobacterium
g__Bacteroides
g__
g__Granulicatella
g__Staphylococcus
g__Blautia
g__Bacteroides
g__Staphylococcus
g__Clostridium
g__Streptococcus
g__Actinomyces
g__Klebsiella
g__
Genus
37b 50A 16A 2A 30A 43a 50C 23B 17A 21A 25B 1B 41A 16B 84b 40B 16C 28a 39C 3C 38C 37B 3B 35B 22B 27C 26C 32C 27B 36b 52A 44C 34B G2 42B 37a 1A G21 G18 43c G5
Sample
Abundance
2.500000e−01
1.562500e−02
9.765625e−04
6.103516e−05

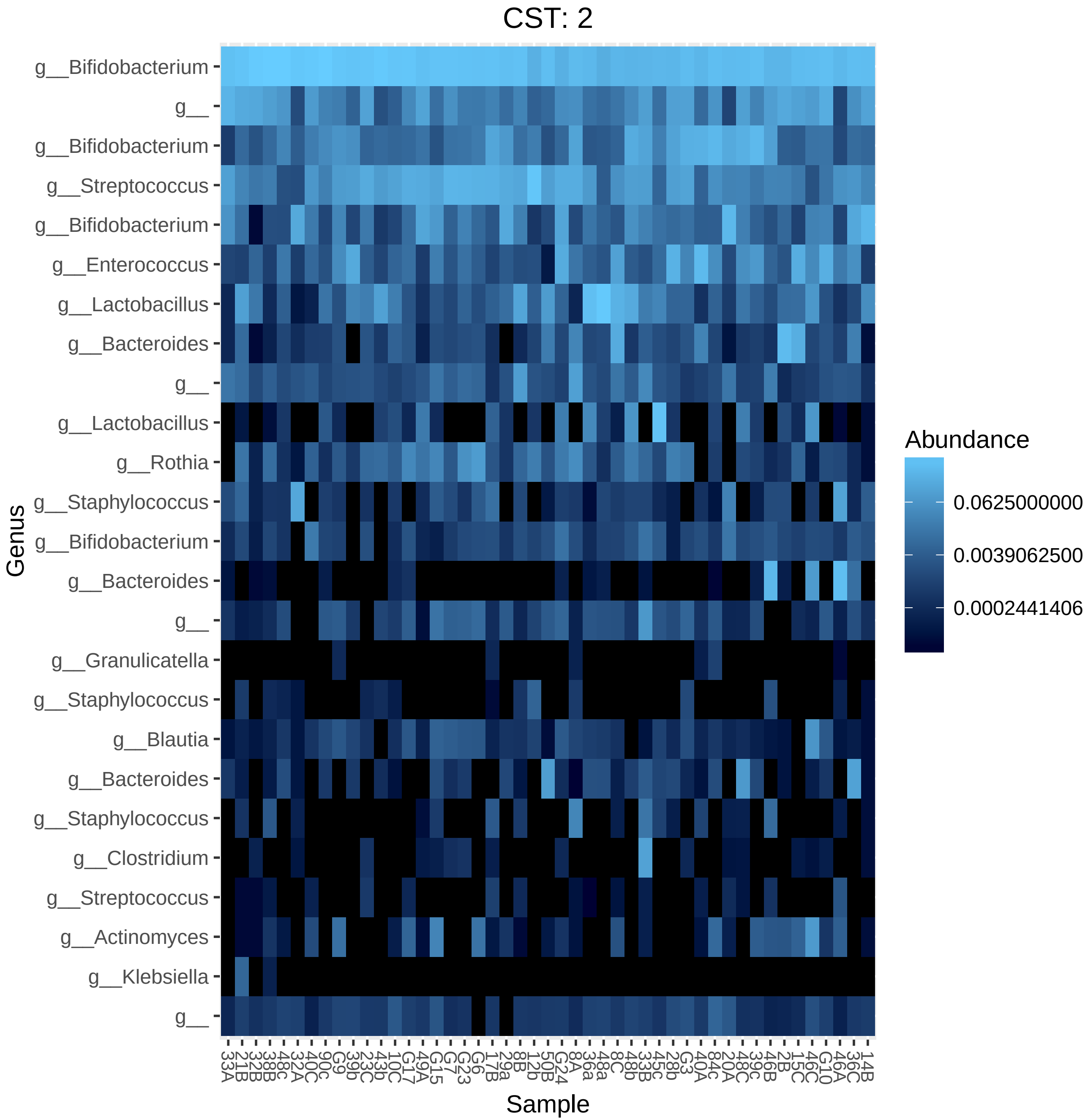
CST: 2
g__Bifidobacterium
g__
g__Bifidobacterium
g__Streptococcus
g__Bifidobacterium
g__Enterococcus
g__Lactobacillus
g__Bacteroides
g__
g__Lactobacillus
g__Rothia
g__Staphylococcus
g__Bifidobacterium
g__Bacteroides
g__
g__Granulicatella
g__Staphylococcus
g__Blautia
g__Bacteroides
g__Staphylococcus
g__Clostridium
g__Streptococcus
g__Actinomyces
g__Klebsiella
g__
Genus
33A 21B 32B 38B 48c 32A 40C 90c G9 39b 23C 43b 10C G17 49A G15 G7 G23 G6 17B 29a 8B 12b 50B G24 8A 36a 48a 8C 48b 33B 45c 28b G3 40A 84c 20A 48C 39c 46B 2B 15C 46C G10 46A 36C 14B
Sample
Abundance
0.0625000000
0.0039062500
0.0002441406

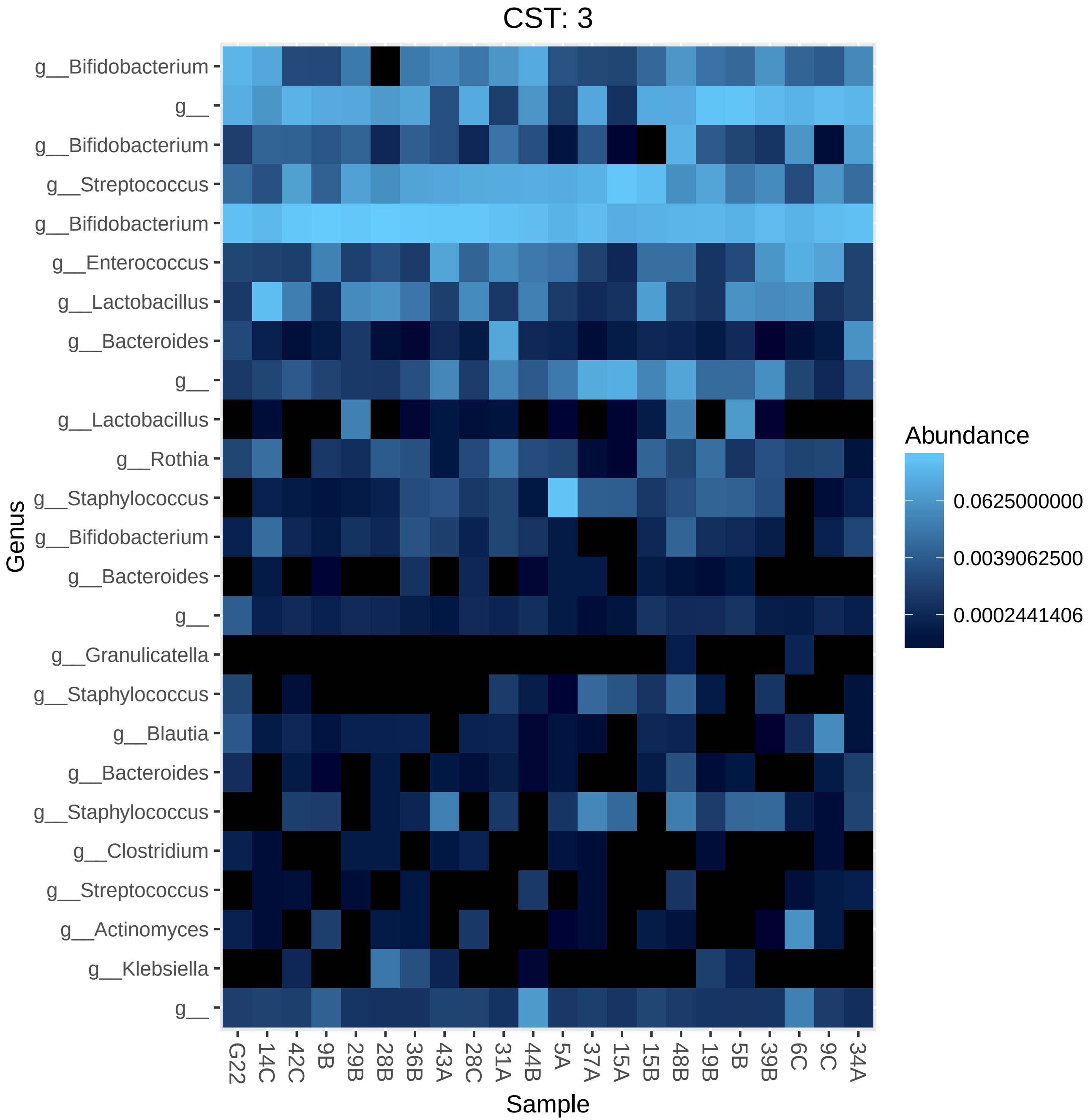
CST: 3
Genus
g__Bifidobacterium
g__
g__Bifidobacterium
g__Streptococcus
g__Bifidobacterium
g__Enterococcus
g__Lactobacillus
g__Bacteroides
g__
g__Lactobacillus
g__Rothia
g__Staphylococcus
g__Bifidobacterium
g__Bacteroides
g__
g__Granulicatella
g__Staphylococcus
g__Blautia
g__Bacteroides
g__Staphylococcus
g__Clostridium
g__Streptococcus
g__Actinomyces
g__Klebsiella
g__
Sample
G22
14C
42C
9B
29B
28B
36B
43A
28C
31A
44B
5A
37A
15A
15B
48B
19B
5B
39B
6C
9C
34A
Abundance
0.0625000000
0.0039062500
0.0002441406

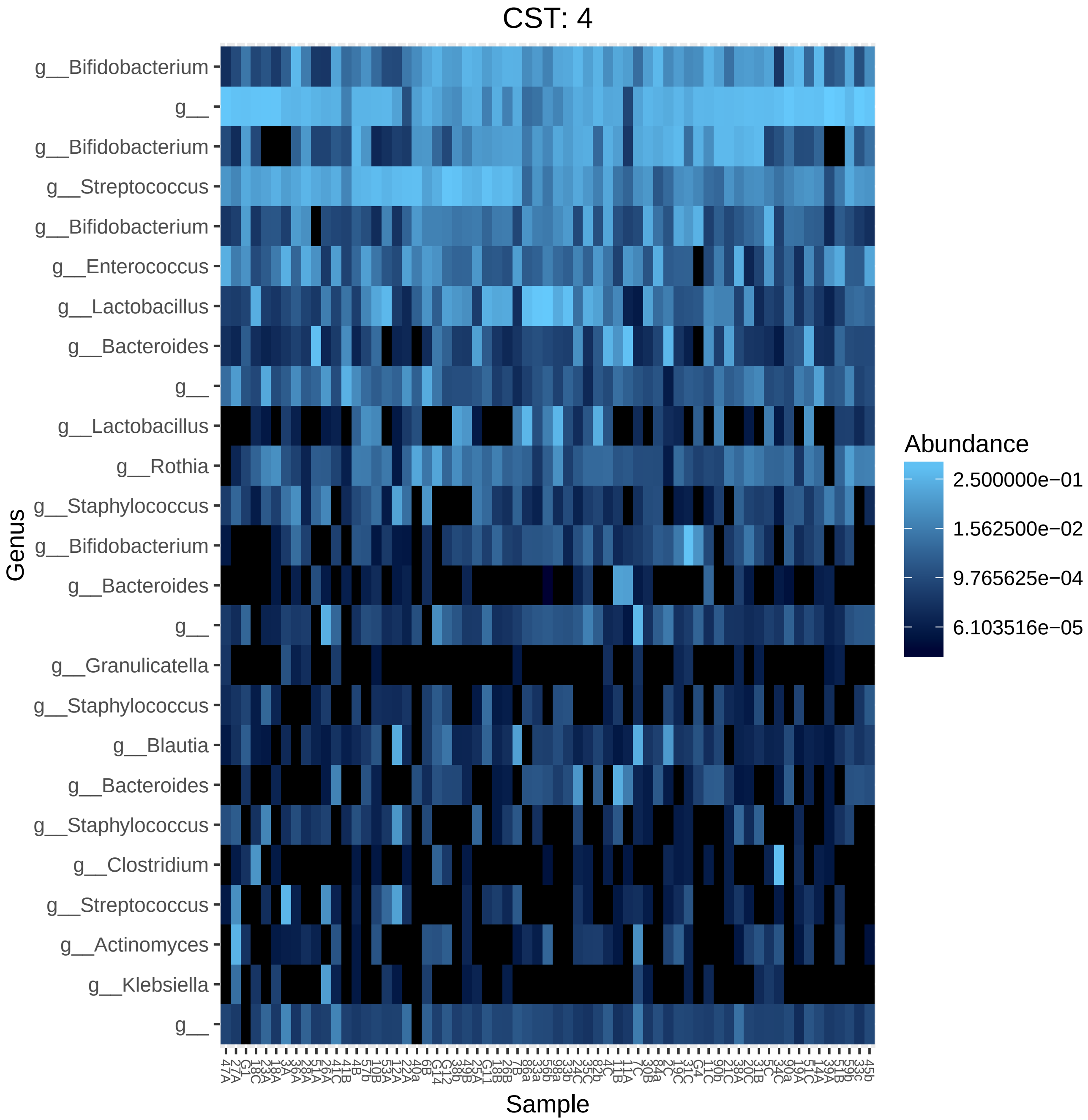

CST: 4
Genus
g__Bifidobacterium
g__
g__Bifidobacterium
g__Streptococcus
g__Bifidobacterium
g__Enterococcus
g__Lactobacillus
g__Bacteroides
g__
g__Lactobacillus
g__Rothia
g__Staphylococcus
g__Bifidobacterium
g__Bacteroides
g__
g__Granulicatella
g__Staphylococcus
g__Blautia
g__Bacteroides
g__Staphylococcus
g__Clostridium
g__Streptococcus
g__Actinomyces
g__Klebsiella
g__
47A 27A G1 18C 23A 18A 3A 36A 28A 51A 26A 41C 41B 4B 57b 10B 53A 12A 22A 40a 6B G14 G12 38b 49B 25A G11 18B 26B 7B 86a 33a 56b 98a 33b 24C 35C 82b 4C 11B 11A 7C 30B 84a 2C 19C 31C G4 11C 90b 21C 38A 20C 31B 5C 34C 90a 19A 51C 14A 39A 51B 59b 33c 45b
Sample
Abundance
2.500000e−01
1.562500e−02
9.765625e−04
6.103516e−05

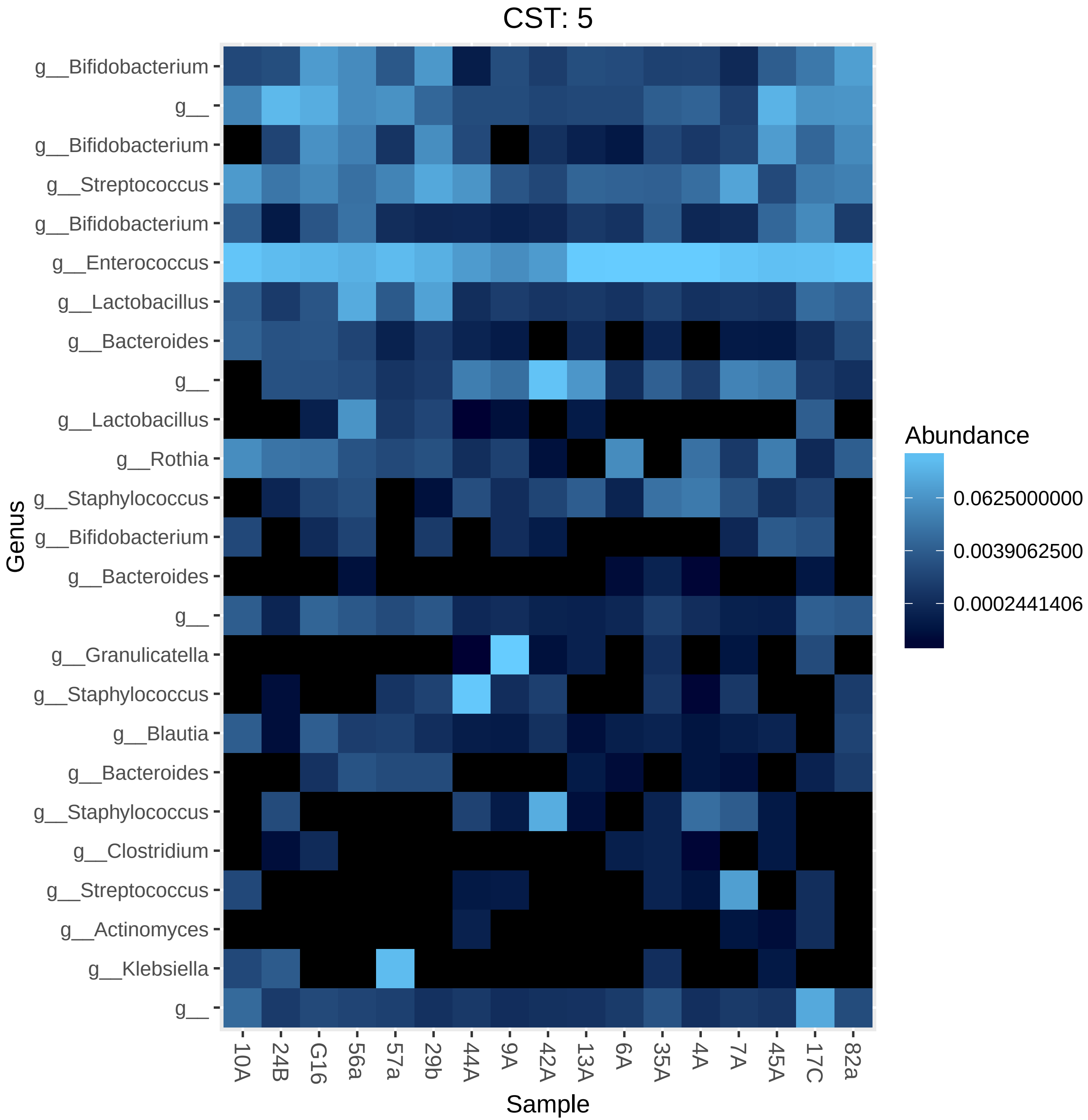
CST: 5
g__Bifidobacterium
g__
g__Bifidobacterium
g__Streptococcus
g__Bifidobacterium
g__Enterococcus
g__Lactobacillus
g__Bacteroides
g__
g__Lactobacillus
g__Rothia
g__Staphylococcus
g__Bifidobacterium
g__Bacteroides
g__
g__Granulicatella
g__Staphylococcus
g__Blautia
g__Bacteroides
g__Staphylococcus
g__Clostridium
g__Streptococcus
g__Actinomyces
g__Klebsiella
g__
Genus
10A
24B
G16
56a
57a
29b
44A
9A
42A
13A
6A
35A
4A
7A
45A
17C
82a
Sample
Abundance
0.0625000000
0.0039062500
0.0002441406
